# Characterization of a Mannose-6-Phosphate Isomerase from *Bacillus amyloliquefaciens* and Its Application in Fructose-6-Phosphate Production

**DOI:** 10.1371/journal.pone.0131585

**Published:** 2015-07-14

**Authors:** Sujan Sigdel, Ranjitha Singh, Tae-Su Kim, Jinglin Li, Sang-Yong Kim, In-Won Kim, Woo-Suk Jung, Cheol-Ho Pan, Yun Chan Kang, Jung-Kul Lee

**Affiliations:** 1 Department of Chemical Engineering, Konkuk University, 1 Hwayang-Dong, Gwangjin-Gu, Seoul 143–701, Republic of Korea; 2 BioNgene Co., Ltd, 10–1 Myungryun-Dong, Chongro-Gu, Seoul 110–521, Republic of Korea; 3 Functional Food Center, Korea Institute of Science and Technology Gangneung Institute, Gangneung 210–340, Republic of Korea; 4 Department of Materials Science and Engineering, Korea University, Anam-Dong, Seongbuk-Gu, Seoul 136–713, Republic of Korea; Virginia Tech, UNITED STATES

## Abstract

The *BaM6PI* gene encoding a mannose-6-phosphate isomerase (M6PI, EC 5.3.1.8) was cloned from *Bacillus amyloliquefaciens* DSM7 and overexpressed in *Escherichia coli*. The enzyme activity of BaM6PI was optimal at pH and temperature of 7.5 and 70°C, respectively, with a *k_cat_/K_m_* of 13,900 s^-1^ mM^-1^ for mannose-6-phosphate (M6P). The purified BaM6PI demonstrated the highest catalytic efficiency of all characterized M6PIs. Although M6PIs have been characterized from several other sources, BaM6PI is distinguished from other M6PIs by its wide pH range and high catalytic efficiency for M6P. The binding orientation of the substrate M6P in the active site of BaM6PI shed light on the molecular basis of its unusually high activity. BaM6PI showed 97% substrate conversion from M6P to fructose-6-phosphate demonstrating the potential for using BaM6PI in industrial applications.

## Introduction

Mannose-6-phosphate isomerases (M6PIs, EC 5.3.1.8) are metal-dependent isomerases that interconvert β-d-mannose-6-phosphate (M6P) to d-fructose-6-phosphate (F6P) in eukaryotes and prokaryotes [1]. Based on enzymatic characterization and sequence alignments, these enzymes have been classified into three types, Type I, Type II, and Type III enzymes [2]. Type I enzymes include zinc-dependent M6PIs and are homologous monofunctional enzymes, catalyzing a single isomerization reaction [3, 4]. Type II enzymes are bifunctional and have limited sequence similarity to Type I enzymes except for a very short motif [1]. Type III enzymes catalyze reversible isomerization reactions but share little identity with sequences of the Type I and Type II enzymes.

M6PI has a significant role in supplying guanosine diphosphate- d-mannose (GDP-d-mannose), the precursor of different mannosylated structures including components of fungal cell wall, bacterial exopolysaccharides [5, 6], GDP-d-rhamnose, and GDP-l-fucose [6, 7]. F6P is a precursor of GDP-l-rhamnose and GDP-l-fucose. All of these nucleotide sugars are important for the synthesis of various antibiotics including bleomycin, nystatin, hygromycin A, pimaricin, ampotericin and candicidin [8].

M6PIs have wide substrate scopes, catalyzing reactions with several different substrates, including the isomerization of rare sugars, which are sugars present in small amounts in nature. Isomerases are of increasing significance for biotechnology applications and are important in the production of rare sugars [9], including l-ribose, l-ribulose, l-tagatose, l-xylulose. These carbohydrates are of proven significance in the food and nutritional industries as sweeteners. They are also important in pharmaceutical industries as drugs against HIV and cancers [10]. As described in detail previously, l-sugar derivatives can be also useful in diabetes treatment as inhibitors of glucose reabsorption [11].

Because M6PI is highly selective for its natural substrate M6P, the conversion of M6P to F6P is efficient. F6P is a phosphorylated chiral sugar that is important in the non-oxidative pentose phosphate pathway and of particular interest for the study of various metabolic diseases [12]. F6P is also an important precursor for the production of d-fructose-1,6-diphosphate via phosphofructokinase 1 catalysis [13]. d-fructose-1,6-diphosphate has proven therapeutic potential; clinical data show that this carbohydrate may be useful in a variety of ischaemic conditions, reduction of ischaemic injury in sickle cell anemia, congestive heart failure, bypass surgery, myocardial infarction, and organ preservation for transplantation [14, 15]. Other important molecules such as glyceraldehyde-3-phosphate and dihydroxyacetone phosphate have medicinal value (candidate drugs for the treatment of degenerative diseases) and can be obtained in a cascade reaction from fructose-1,6-diphosphate with the help of fructose 1,6-bisphosphate aldolase [12, 16]. Both enzymatic and chemical processes have been developed for large-scale production of M6P using inexpensive starting materials such as d-mannose and methyl-d-mannoside [17, 18]. Enzymatic production includes phosphorylation of mannose by hexokinase [18], and chemical synthesis includes a simple three-step reaction that converts methyl-d-mannoside to M6P [17] (S1 Fig). There remains a great and urgent need for an industrially applicable and efficient method for the enzymatic synthesis of F6P. Fructose-6-phosphate aldolase is required for a key step in a complicated multi-enzymatic cascade reaction for the synthesis of F6P, which simultaneously utilizes three enzyme systems, and a donor and acceptor substrate [12, 19]. Compared to this process, large amounts of M6P were obtained as an intermediate product from the enzymatic (hexokinase) [18] and chemical synthesis pathways [17], which utilized cheaper substrates; therefore, this could be more efficient for the synthesis of F6P using M6PI.

Type I M6PI catalyzes an aldose-ketose isomerization in which a hydrogen atom moves between C1 and C2 of the aldose or ketose following a *cis*-enediol mechanism as proposed by Roux et al. [20]. The reaction was initiated by M6P binding; the opening of the ring was assisted by the water bound to the zinc and Gln residue, which was followed by conformational change and water displacement [20]. Subsequently, the compound was isomerized with the abstraction of hydrogen at C2 in M6P, and by the protonation of the C1 carbon atom, with the aid of the catalytically important Lys and Glu residues. The linear product F6P was formed at the end; the entry of a new water molecule resulted in ring closure and the subsequent release of the product (F6P; S2 Fig). Crystal structures for the Type I M6PIs from *Candida albicans* [21] (PDB ID 1PMI), *Bacillus subtilis* (PDB ID 1QWR), *Salmonella typhimurium* [22] (PDB ID 2WFP), and *Archaeoglobus fulgidus* (PDB ID 1ZX5) have been reported. Recent studies have shown the characterization of M6PIs from different sources, such as *Thermus thermophilus* [23], *Bacillus subtilis* [24], humans [2], *Candida albicans* [2], *Salmonella typhimurium* [22], and *Saccharomyces cerevisiae*[25]. However, F6P has not been efficiently produced by bioconversion or metabolic engineering because of several reasons, including the low catalytic efficiency of enzymes involved. Therefore, M6PI with a high catalytic efficiency must be discovered or developed. Although Type I M6PIs from various microorganisms have been characterized, and the mechanism of isomerization has been proposed, M6PI from *Bacillus amyloliquefaciens* (BaM6PI) has not been previously reported. Herein we have reported characterization of recombinant BaM6PI together with the production of F6P by recombinant *Escherichia coli* harboring the BaM6PI-encoding gene. We provide experimental data to show that BaM6PI is an M6PI selective for M6P and the most active M6PI reported in the literature. The cloning, heterologous expression, and activity of M6PI from *B*. *subtilis* (BsM6PI) are also reported. The crystal structure of BsM6PI was previously deposited in the protein data bank (PDB ID 1QWR). The molecular interactions and binding orientation of the substrate M6P in the active site of BaM6PI were examined and compared with the crystal structure of BsM6PI.

## Materials and Methods

### Materials

pGEM-T Easy vector, DNA polymerase and DNA extraction kit were obtained from Promega (Madison, WI, USA). Plasmid isolation kit, pET28a expression vector, and nickel-nitrilotriacetic acid (NiNTA) superflow column were purchased from Qiagen (Hilden, Germany). The restriction enzymes (*Bam*HI and *Sal*I) and T4 DNA ligase were purchased from New England Biolabs (Ipswich, USA). And all other chemicals for assays were obtained from Sigma-Aldrich (St. Louis, MO, USA).

### Strains and general culture conditions

*B*. *amyloliquefaciens* DSM 7 (KCCM 40765) and *B*. *subtilis* strain 168 (ATCC 23857) were purchased from the Korean Culture Center of Microorganisms (KCCM) and American Type Culture Collection (ATCC), respectively. *B*. *amyloliquefaciens* and *B*. *subtilis* were grown in Difco nutrient broth (Becton, Dickinson and Company, Franklin Lakes, NJ, USA) at 37°C (pH 6.8). *E*. *coli* DH5α and *E*. *coli* BL21 (DE3) from Invitrogen (Carlsbad, CA, USA) were used for plasmid transformation and expression, respectively. Luria-Bertani medium containing 100 μg ml^-1^ of ampicillin was used to grow *E*. *coli* strains.

### Cloning and expression of the M6PI-encoding gene from *B*. *amyloliquefaciens* and *B*. *subtilis*

PCR was performed to amplify the M6PI-encoding genes from *B*. *amyloliquefaciens* and *B*. *subtilis* using two oligonucleotide primers, 5′- GGATCC ATGACGAAGCTCTTATTTTTAG-3′ (underlined is the *Bam*HI site); 5′- GTCGAC TTAAGATGATGAAGGATGTGAA -3′ (underlined is the *Sal*I site) and 5′- GGATCC ATGACGCAATCAC-3′ (underlined is the *Bam*HI site); 5′- GTCGAC TTAAATATGAGACACGAT-3′ (underlined is the *Sal*I site). The *Bam*HI and *Sal*I sites were used for subcloning into pET28a expression vector. Cloning of M6PI- encoding genes and expression of recombinant BaM6PI and BsM6PI were performed as described in detail previously [26]. The *M6PI* genes released from the pGEM-T vector were ligated with the pET28a vector to give pET-M6PI, and BaM6PI or BsM6PI was expressed as a fusion at the N terminus to a His_6_ tag. The enzyme expression was induced using 0.05 mM isopropyl-β-d-thiogalactopyranoside (IPTG). The cells were then harvested by centrifugation at 10,000 g (4°C, 15 min) and washed (20 mM phosphate-buffered saline, pH 7).

### Purification of recombinant M6PIs and determination of molecular mass

As described in detail previously, purification of recombinant BaM6PI and BsM6PI was performed using NiNTA agarose [27]. The purified enzyme fractions were then analyzed by SDS-PAGE according to the previous report [28]. Concentration of the proteins were determined using the Bradford assay [29, 30]. Bovine serum albumin was used as a standard protein. To determine the molecular mass, gel filtration was used as described previously [31]. Ribonuclease A (13.7 kDa), ovalbumin (44 kDa), conalbumin (75 kDa), and aldolase (158 kDa) were used as reference proteins (Amersham, UK) [32]. SDS-PAGE was used to estimate subunit molecular mass under denaturing conditions, using a prestained marker (Bio-Rad) with reference proteins.

### Enzyme assay

M6PI activity was measured by determining the amount of F6P formed per unit time. Under standard conditions, a reaction mixture contained 0.5 mM CoCl_2_, ~1 ng of enzyme, 15 mM M6P (substrate), and 20 mM phosphate buffer pH 7.5, in a final volume of 100 μl. The reaction mixture was kept at 70°C for 5 min followed by cooling on ice to slow the reaction. The product F6P was then quantitated by the cysteine carbazole sulfuric acid method [33]. Isomerization of the substrate and accumulation of the product in the reaction mixture were also estimated by HPLC (Ultimate 3000 series, Thermo Scientific, USA) using analytical column NH2P-50 4E (Shodex) at 30°C, a mobile phase of 75% acetonitrile in water.

### Physicochemical property

The optimum temperature of the enzyme samples were assayed for 5 min at varying temperatures (30–80°C). The optimum pH was determined using five different buffer systems, glycine-HCl buffer (20 mM, pH 3–4), sodium acetate buffer (20 mM, pH 4–6), phosphate buffer (20 mM, pH 6–8), Tris–HCl buffer (20 mM, pH 8–9) and glycine-NaOH (20 mM, pH 9–11) buffer. Enzyme thermostability was also assessed at various temperatures using 20 mM phosphate buffer with 0.5 mM Co^2+^.

### Effect of metal ions on M6PI activity

Dialysis of the purified BaM6PI was performed using 20 mM phosphate buffer (pH 7.5) containing 10 mM EDTA as described previously [34]. Next, in the presence of 0.5 mM metal ions (MnCl_2_, MgCl_2_, ZnCl_2_, CoCl_2_, FeSO_4_, CaCl_2_, BaCl_2_, CuSO_4_, and KCl), enzyme assay was performed under standard conditions.

### Determination of kinetic parameters

The kinetic constants were determined in 20 mM phosphate buffer (pH 7.5), 0.5 mM Co^2+^, and 1–30 mM substrate (M6P) at 70°C. Using the Michaelis-Menten equation in Prism 5 (Graphpad Software, CA, USA), *V*_*max*_ (U/mg protein) and *K*_*m*_ (mM) values were determined.

### Enzymatic F6P production

The production of F6P catalyzed by BaM6PI was studied in 15-ml round bottom tubes, each containing a total reaction volume of 1 ml. The reaction mixture contained 80 mM borate buffer pH 9.0, 0.5 mM Co^2+^, and 50 μg of BaM6PI; the reaction was performed at 40°C with shaking at ~180 rpm. To start the reaction, 100 mM M6P (substrate) was added. Samples were withdrawn at constant intervals and analyzed using the cysteine-carbazole method [33], measuring the absorbance at 560 nm. F6P production was also determined by HPLC (Ultimate 3000 series) [35]. The HPLC system was equipped with an evaporative light scattering detector (ELSD) (Chromachem, MA, USA). A Shodex Sugar SP0810 column was used at 80°C for the analysis as described in detail previously [36]. The samples were filtered through a 0.22-μm cellulose acetate membrane before HPLC analysis. F6P standard was purchased from Sigma-Aldrich. For comparative study, production of F6P by BsM6PI was also performed following the same procedure described above.

### Homology modeling and model validation

The three-dimensional model of BaM6PI was generated using the Build Homology Models (MODELER) option in Discovery Studio 3.5 (DS 3.5, Accelrys Software, San Diego, CA, USA). The crystal structure of BsM6PI (PDB ID 1QWR) with sequence identity of 53.4% with the BaM6PI was used as a template. Homology modeling, assessment of the model quality, validation, model refinement, and binding site identification were determined as previously described [37].

### Molecular dynamics (MD) simulation and docking

CDOCKER was used for the docking study [38]. The CHARMm force field was applied for energy minimization and MD simulation as described previously [39, 40]. A solvation protocol was used to create an explicitly solvated system using CHARMm with a 15 Å cap from the center of the mass of BaM6PI as described in detail previously [37]. Catalytic Zn^2+^ was docked into the Zn^2+^ binding site and then an energy-minimized structure was used for the docking of substrate. For the comparative study, substrate was docked in the energy-minimized model of the K96Q mutant protein. The point mutation was created using the build mutants option of DS 3.5. For both the BaM6PI and BsM6PI K96Q models, different substrate conformations were generated, and candidate poses after docking were chosen as described previously [37]. Docking of the substrate into the BaM6PI model was validated by superimposing the model on the substrate-docked K96Q BsM6PI model and the substrate-docked M6PI crystal structure from *C*. *albicans* (PDB ID 1PMI). Catalytic residues were in a conserved geometry in all the cases, and docking of the substrate in the BaM6PI model could therefore be validated. S3 Fig shows the superimposition, where the RMSDs of the model compared with the BsM6PI and 1PMI crystal structures were 1.2 Å and 2.2 Å, respectively. These suggest that the docked Zn^2+^ and substrate, and their interactions with the residues in BaM6PI, are of a similar geometry compared with the 1PMI structure as well as with two additional M6PIs.

## Results and Discussion

### Identification and characterization of the M6PI gene encoding a type I M6PI

Whole genome sequence analysis of *B*. *amyloliquefaciens* DSM 7 (FN597644) suggested the presence of an *M6PI* gene. The open reading frame was annotated as a putative Type I *M6PI*, suggesting that this open reading frame might encode an M6PI catalyzing the conversion of M6P to F6P. The *M6PI* gene encodes 314 amino acids with a molecular mass of 35,492 Da and has 48% of the overall GC content. The deduced gene product, BaM6PI, showed 61%, 57%, 54%, and 37% amino acid identity with M6PIs from *B*. *subtilis* HB002 (Genbank accession number AF324506), *Geobacillus thermodenitrificans* NG80-2 (Genbank accession number CP000557), *B*. *subtilis* 168 (Genbank accession number BAA08088.1), and *Thermus thermophilus* HB8 (Genbank accession number AP008226), respectively. Thus, we considered BaM6PI a candidate M6PI from *B*. *amyloliquefaciens*.

### Heterologous expression and purification of BaM6PI

**Fig 1:**
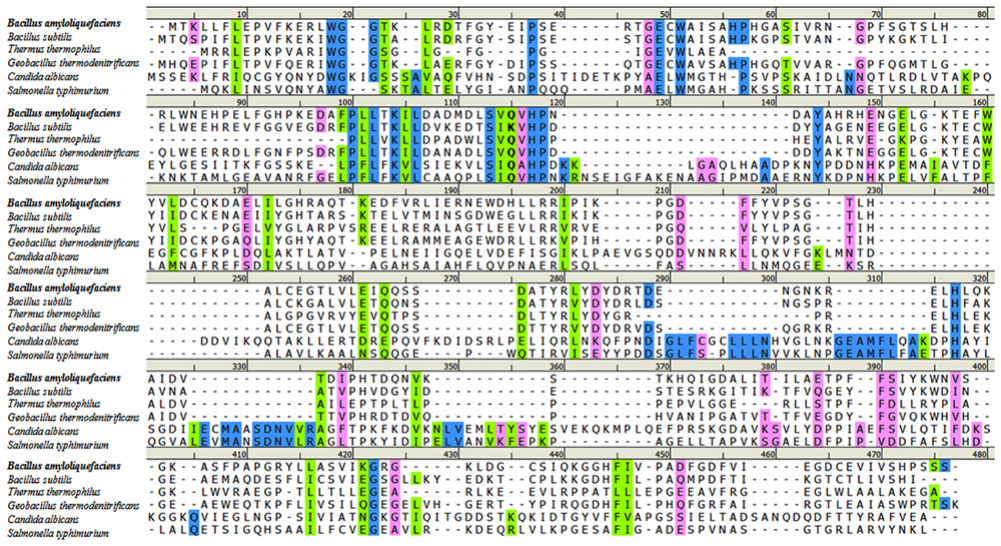
Multiple sequence alignment of M6PIs. Sequences used for alignment are *B*. *amyloliquefaciens* (BaM6PI, this study), *B*. *subtilis* (BsM6PI, PDB ID 1QWR), *T*. *thermophilus*, *G*. *thermodenitrificans*, *C*. *albicans*, and *S*. *typhimurium*. Residues in blue, green, and magenta background denote identical, strong similarity, and weak similarity, respectively. The bold column shows the location of the conserved glutamine, which is a lysine in BsM6PI.

To investigate the proposed function of BaM6PI, the *BaM6PI* gene was cloned into the T7 promoter-based plasmid pET28a to give pET28a-BaM6PI. Then the gene was expressed in *E*. *coli* BL21. The extracts of *E*. *coli* BL21 containing pET28a-BaM6PI showed a high level M6PI activity in comparison with the control *E*. *coli* BL21 containing plasmid pET28a only. To determine if F6P was produced from M6P specifically by BaM6PI and not by other enzymes in the *E*. *coli* host cell because of the overexpression of the *BaM6PI* gene, the BaM6PI enzyme was purified. In SDS-PAGE, a 35-kDa protein, corresponding to the predicted molecular mass of the BaM6PI protein, was identified in the extracts from cells containing pET28a-BaM6PI and that had been induced by IPTG. Isomerization of M6P was assessed by the formation of ketose F6P indicated by changes in the absorbance at 560 nm. These results supported the hypothesis that the M6PI activity shown in the extracts of *E*. *coli* BL21 containing pET28a-BaM6PI corresponded to the activity of the BaM6PI protein. The same process was used with the BsM6PI protein. However, BsM6PI did not catalyze the isomerization of M6P, although it exhibited similar physical properties. The lack of activity of BsM6PI could be due to the substitution of a lysine residue at position 116 with a highly conserved glutamine residue (Fig 1). The sequence alignment showed that a lysine residue was present at position 96 only in *B*. *subtilis*. Upon mutation of the lysine in BsM6PI to glutamine, the K96Q mutant of BsM6PI showed a significant M6PI activity (22,000 μmol min^-1^ mg-protein^-1^).

### Determination of molecular mass and quaternary structure

The subunit molecular mass of BaM6PI was calculated as 35.49 kDa, based on the amino acid sequence using the protparam program of ExPASy Proteomics Server. This is in agreement with the results of SDS-PAGE analysis, which indicated a subunit molecular mass of purified BaM6PI of ~ 35 kDa (S4 Fig). The native molecular mass of BaM6PI was determined to be 35 kDa using gel filtration chromatography on a Sephacryl S-300 HR 16/60 column (S5 Fig), indicating that the enzyme is a monomer. Microbial M6PIs that have been characterized in detail are primarily monomeric [2, 22].

### Optimum pH, temperature, and thermal stability

**Fig 2:**
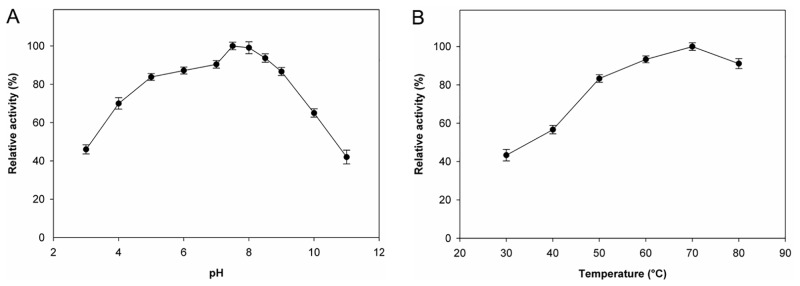
Effect of (A) pH and (B) temperature on BaM6PI activity. Enzyme reactions were performed under standard conditions in the presence of 15 mM M6P. Activities at the optimal temperature and pH were defined as 100%. Each data represents the mean of three measurements with standard deviation of not more than 10%.

The effect of pH and temperature on BaM6PI was investigated by measuring the activities of BaM6PI reactions at pH 3–11 and at temperatures from 30 to 80°C. The pH optimum for isomerization of M6P by purified BaM6PI was 7.5, with 83%, 84%, 87%, 90%, 99%, 94%, and 86% of the activity at pH 4.0, 5.0, 6.0, 7.0, 8.0, 8.5, and 9.0, respectively (Fig 2A). The pH-range of the BaM6PI activity was broad, with relative activity greater than 85% from pH 5.0 to 9.0. The optimum temperature for BaM6PI was 70°C (Fig 2B). BaM6PI with 0.5 mM Co^2+^ was stable at 40°C with half-life of 15 h. Thermal stability of BaM6PI decreased with the increase of temperature; half-lives at 50°C, 60°C and 70°C were 3.5 h, 10 min and 8 min, respectively.

### Metal ion effects on BaM6PI activity

The enzyme was purified without added metal ions as described in Methods, and then dialyzed with 10 mM EDTA. BaM6PI activity was lost by EDTA and was restored upon addition of Co^2+^, Zn^2+^, or Mg^2+^. BaM6PI showed the maximum restoration of activity in the presence of Co^2+^. Co^2+^ at 0.5 mM was the most effective metal ion for isomerization of M6P, resulting in approximately 1.7-fold increase in activity, relative to the control (S6 Fig). Thus, 0.5 mM concentration of metal ion was used for the study. The sequence alignment suggested the presence of conserved metal binding residues Gln98, His175, His100 and Glu118. A number of divalent metal ions, such as Co^2+^, Mn^2+^, Zn^2+^, Fe^2+^, Cu^2+^, and Mg^2+^, restored the activity of EDTA-treated zinc metalloenzymes [41]. BaM6PI contains one catalytic zinc atom per monomer as inferred from inductively coupled plasma (ICP)-MS analysis. Typically, M6PI requires a divalent metal cofactor such as Zn^2+^ for activity [6].

### Substrate selectivity

The functional assignment of BaM6PI as an M6PI allowed us to further investigate its substrate specificity towards aldoses and ketoses. M6P, l-talose, l-ribose, l-ribulose, l-lyxose, l-allose, d-ribose, d-allose, and l-mannose were used to examine the substrate selectivity of BaM6PI. BaM6PI had a very high preference for M6P compared with other substrates (Table 1). BaM6PI also showed activity with l-ribulose, l-talose, and d-ribose to give products l-ribose, l-tagatose, and d-ribulose, respectively (Table 1).

**Table 1:** Specific activity of mannose-6-phosphate isomerase from *B*. *amyloliquefaciens* for various substrates.

Substrate	Product	Specific activity (μmol min^-1^ mg-protein^-1^)
d-Mannose-6-phosphate	d-Fructose-6-phosphate	81,000
l-Ribulose	l-Ribose	24
l-Lyxose	l-Xylulose	2.0
l-Ribose	l-Ribulose	7.2
l-Talose	l-Tagatose	14
d-Ribose	d-Ribulose	31
l-Allose	l-Psicose	1.0
l-Mannose	l-Fructose	11
d-Allose	d-Psicose	2.1

### Kinetics parameters

**Fig 3:**
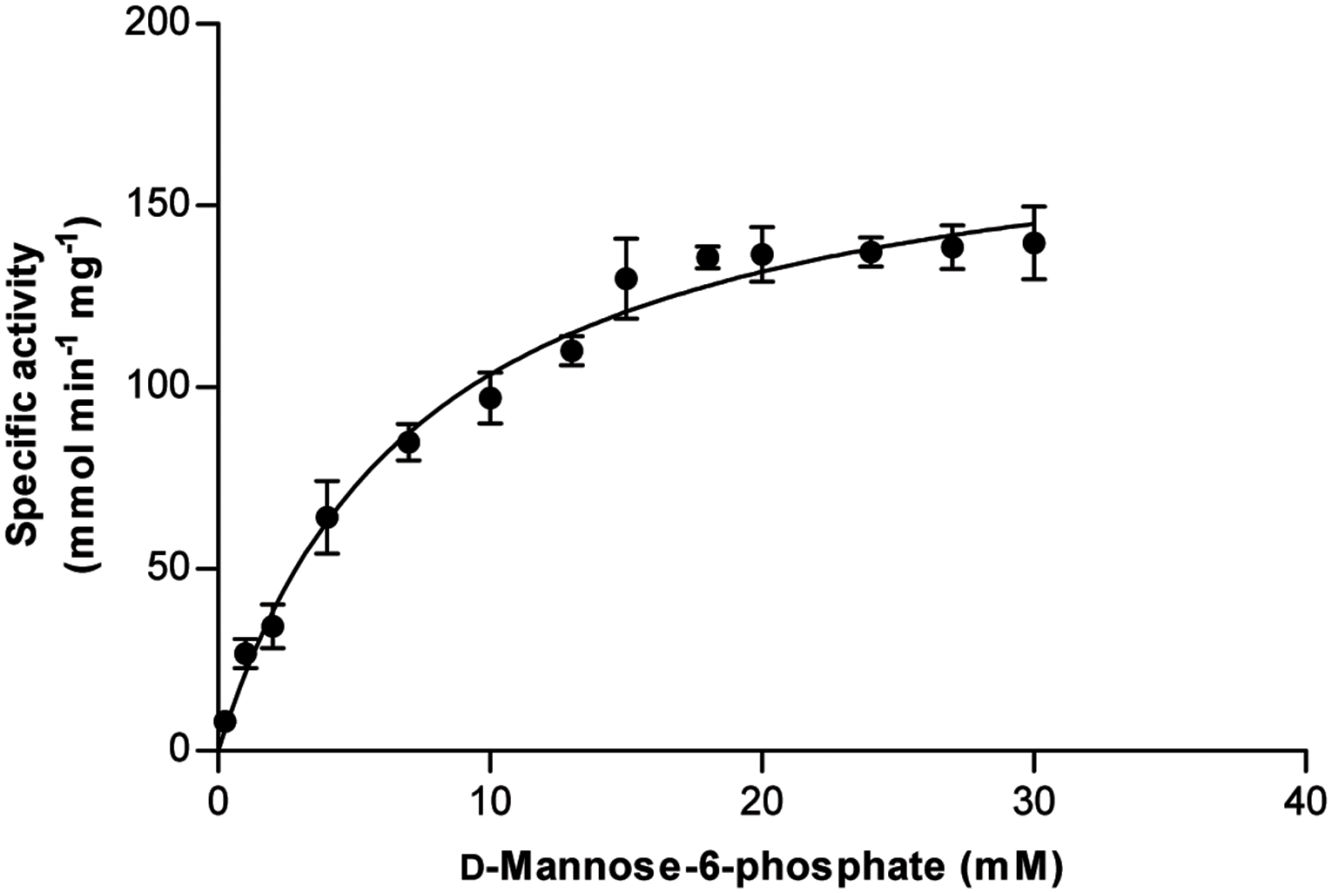
Effect of M6P concentration on BaM6PI activity. The enzyme activity was assayed with varying concentration of M6P at pH 7.5. Each data represents the mean of three measurements with standard deviation of not more than 10%.

Under standard assay conditions at pH 7.5, initial velocities were determined. M6P as a substrate provided a hyperbolic curve and a linear double-reciprocal plot. M6P concentration was varied from 0.25 to 30 mM. Fig 3 shows a typical Michaelis-Menten behavior for BaM6PI activity. A near-maximum enzyme activity (initial velocity approaching *V*_*max*_) was obtained with an M6P concentration of approximately 15 mM under the experimental conditions. The non-linear regression fitting of experimental data obtained for M6P conversion to the Michaelis-Menten kinetic equation showed that *k*_*cat*_, *K*_*m*_, and *k*_*cat*_ */K*_*m*_ for M6P were 106,000 s^-1^, 7.62 mM, and 13,900 s^-1^ mM^-1^, respectively. As described above, BsM6PI was not active, but K96Q mutant of BsM6PI showed a significant M6PI activity. At an optimized temperature of 50°C and phosphate buffer pH 7.5, *K*_*m*_, *k*_*cat*_, and *k*_*cat*_ */K*_*m*_ for M6P were 12 mM, 50,300 s^-1^, and 4,190 s^-1^ mM^-1^, respectively. Table 2 shows a comparison of the properties of various M6PIs from different sources. Notably, BaM6PI has the highest catalytic efficiency of all characterized M6PIs. We focused on finding an M6PI enzyme that showed high activity towards its natural substrate M6P, which would produce F6P as a value-added product. For this purpose, putative M6PI genes were screened from 26 different bacterial sources (S1 Table), including *Bacillus amyloliquefaciens* DSM7 (this study), *Bacillus subtilis* (this study), *Bacillus licheniformis*, *Streptomyces venezuelae*, and *Lactobacillus salivarius*. Among these, the M6PI from *Bacillus amyloliquefaciens* DSM7 (BaM6PI) was observed to be highly active and specific to M6P under a wide range of pH, compared to other previously reported and screened M6PIs.

**Table 2:** Biochemical and kinetic properties of M6PIs from various organisms for mannose-6-phosphate.

Organism	*M*_*r*_, subunit (kDa)	Optimum pH	*K*_*m*_ (mM)	*k*_*cat*_ (s^-1^)	*k*_*cat/*_ *K*_*m*_ (s^-1^ mM^-1^)	References
*Thermus thermophilus*	29	7	0.21	1,371	6,685	[23]
*Bacillus subtilis*	36.5	7.5	29	22,730	770	[24]
*Human* M6PI	46	8	0.25	84	337	[2]
*Candida albicans*	48	8.5	1.24	960	774	[2]
*Saccharomyces cerevisiae*	48	8	0.65	784	1206	[25]
*Salmonella typhimurium*	42	8.5	1.34	583	435	[22]
*Bacillus amyloliquefaciens*	35	7.5	7.62	106,000	13,900	This study

### Enzymatic F6P production

**Fig 4:**
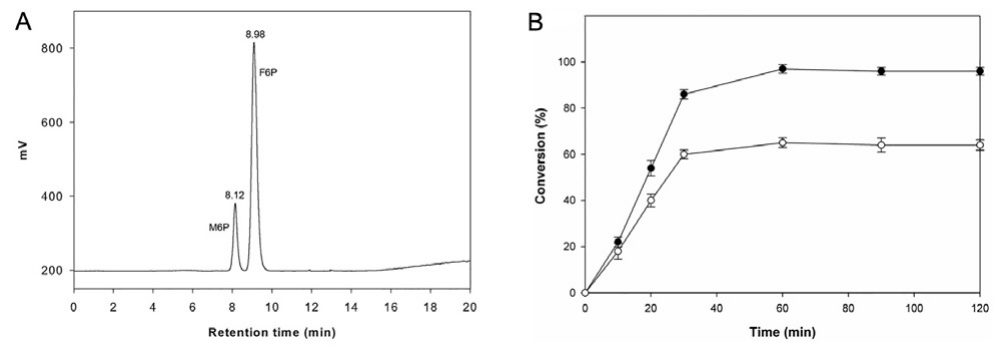
Production profile of F6P. (A) HPLC chromatogram of the reaction mixture after 25 mins of the reaction; both substrate (M6P) and product (F6P) are seen. (B) Conversion profile of substrate (M6P) to the product (F6P) in phosphate buffer (open circle) and in borate buffer (filled circle).

F6P is an important phosphorylated carbohydrate involved in the non-oxidative pentose phosphate pathway and is of particular interest for the study of various metabolic diseases. F6P is also an important precursor for the production of d-fructose-1,6-diphosphate and various other phosphate sugars with important therapeutic values for the treatment of sickle cell anemia, congestive heart failure, myocardial infarction, and neurodegenerative disease [14, 15]. To obtain F6P in a sufficiently large quantity, a less expensive high-yield production method must be developed. Large-scale enzymatic and synthetic procedures for M6P have already been developed, using relatively inexpensive starting materials such as d-mannose and methyl-d-mannoside. In this study, enzymatic procedure using the purified BaM6PI was utilized for the isomerization of M6P to produce F6P (S4 Fig). The F6P product coeluted with an authentic standard of F6P, and no other peak was found indicating that no byproducts formed in the reaction (Fig 4A). The conversion of M6P to F6P reached 97% after 1 h at 40°C in borate buffer. In the absence of borate, only 64% conversion was obtained (Fig 4B).

**Fig 5:**
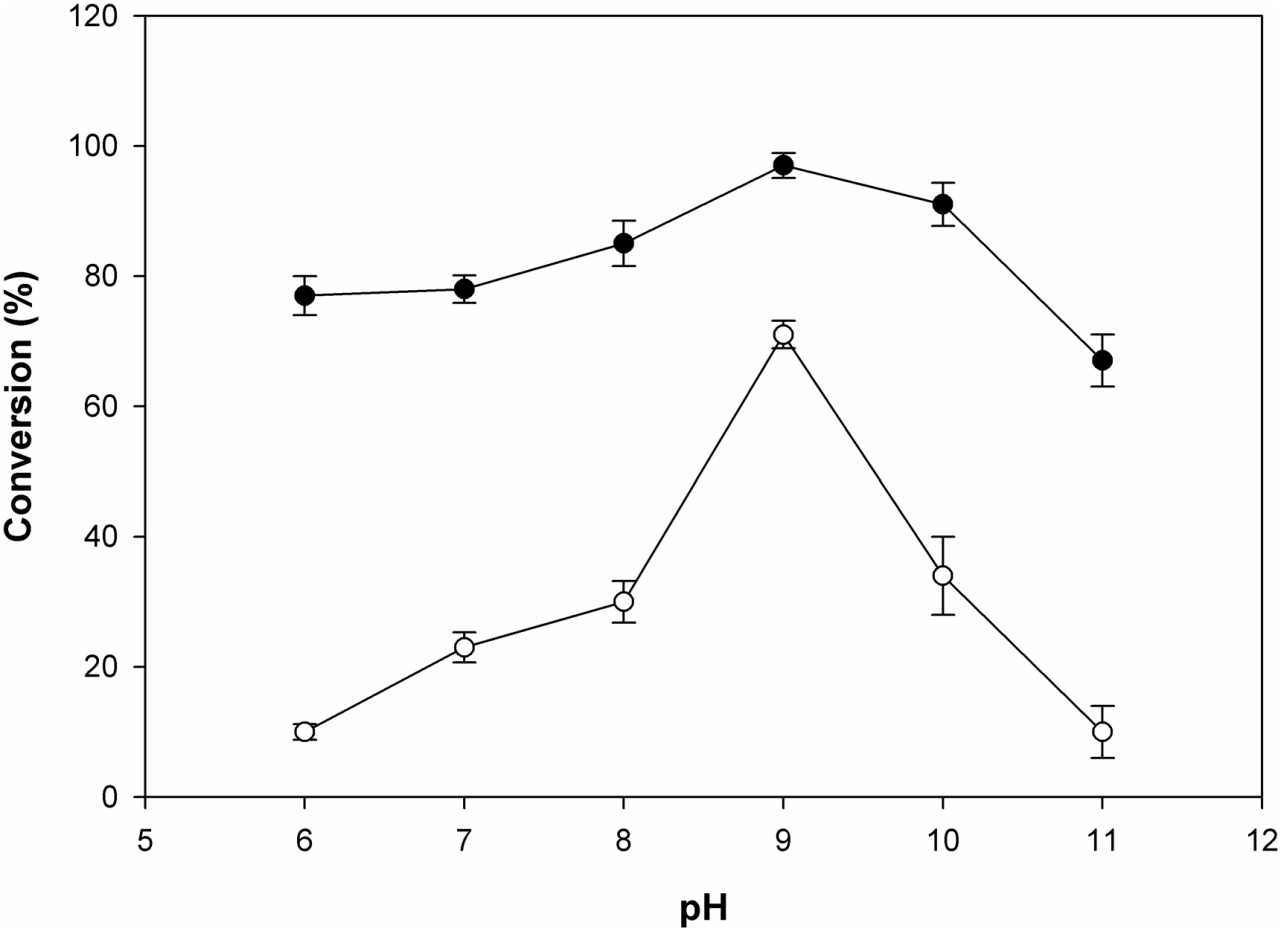
Effect of pH on the conversion of M6P to F6P in the presence of borate. Filled circle BaM6PI, open circle BsM6PI. M6P 100 mM, temperature 40°C, time 1 h, borate concentration 80 mM.

For the comparative study, conversion of M6P to F6P in the presence of borate buffer (80 mM) over a pH range from 6–10 was checked for both BaM6PI and BsM6PI. At pH 9, BaM6PI achieved 97% conversion, whereas BsM6PI achieved only 71% conversion (Fig 5). BaM6PI showed a wide range of activity from pH 6–9 (more than 80% at pH 9) (S7 Fig), resulting in higher conversion at pH 9 in borate buffer. However, BsM6PI had comparatively lower activity at pH values above 8 (43% at pH 9) (S7 Fig), leading to lower conversion at pH 9, even in the presence of borate.

BaM6PI exhibits high activity over a wide pH range, making BaM6PI a good choice for industrial applications both at acidic and basic pH [31, 42]. However, M6PIs characterized previously have narrow optimum pHs with a range of 7 to 8.5 [23, 24]. The wide pH range of BaM6PI is an important factor that makes it novel when compared to BsM6PI having narrow pH range (Fig 5). This wide pH range made the higher conversion of M6P to F6P possible at pH 9, demonstrating the importance of a wide pH range for the production of therapeutically important F6P. Browning of the product was not observed in F6P production, since the reaction was performed at a lower temperature of 40°C. In addition, there was no by-product observed, as was confirmed from the HPLC chromatogram as shown in Fig 4A.

Borate was used in the production because a high yield in the chemical isomerization of aldoses to ketoses could be achieved in alkaline solutions containing borate [42]. Generally, with borate buffer, the isomerase and epimerase enzyme activity is higher at alkaline pH (above 8) when compared to a buffer without borate [42–45]. A similar pattern was observed in this case, where higher conversion at pH 9 for both the enzymes was also observed. In the presence of borate, the conversion of M6P to F6P was highly pH-dependent (Fig 5). Boron exists as boric acid or borate ions in aqueous solution [42, 46, 47]. The p*K*_a_ of boric acid is 9.2, and borate ions form complexes with F6P at pH 9. In the current study, the borate concentration increased rapidly with the shift of pH from 8 to 9, allowing formation of the complex between borate and F6P. Thus, the formed F6P-borate complex is not likely to participate in the equilibrium reaction and the result is as if the product was removed. Therefore, more M6P is converted to F6P to restore the equilibrium.

### Homology modeling of BaM6PI

The generated model structure of BaM6PI was improved through subsequent refinement of the loop conformations using the Protein Health module of DS 3.5. To validate the model, different tools were used, including PROCHECK [48]. The calculated Ramachandran plot suggested 89.5%, 9.4%, 0.7%, and 0.4% of the residues in the derived model are in the most favored, additional allowed, generously allowed, and disallowed regions, respectively (S8 Fig). Altogether, 99% of the residues could be placed in the combined favored and allowed regions. The selected template 1QWR had a sequence identity of 53%, and the backbone of the polypeptide chain superimposed on that of the BaM6PI model with an RMSD of 0.43 Å. Conserved catalytic residues in the active site pocket of BaM6PI model had similar orientations as those in the template (PDB ID 1QWR).

### MD simulation and docking

**Fig 6:**
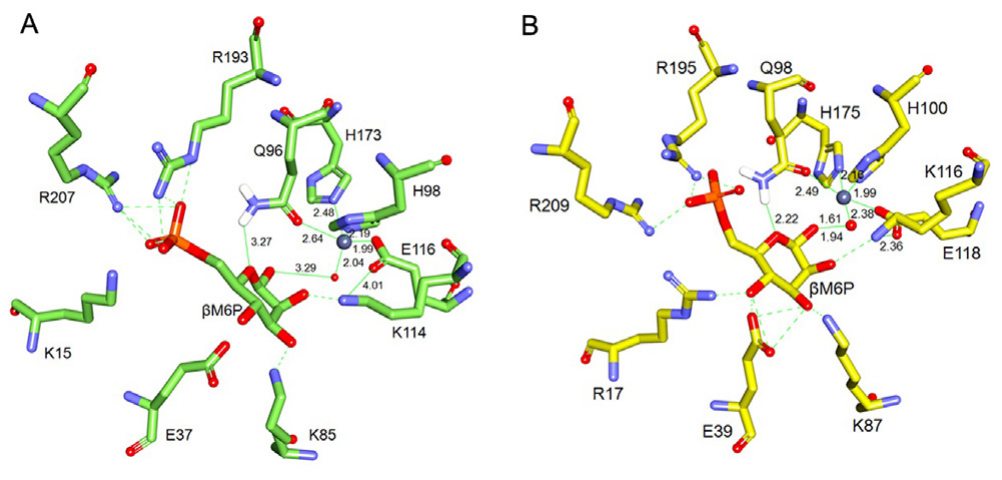
Active sites of the BsM6PI mutated model (A) and BaM6PI model (B) complexed with cyclic substrate β-d-mannose-6-phosphate (M6P). The zinc metal cofactor and water are shown as grey and red spheres, respectively. The green dashed lines indicate hydrogen-bonding interactions. The lengths of the potential hydrogen bonds and other coordinating bonds are shown in Å next to the green dashed and plane lines, respectively.

To propose an explanation for the high M6PI activity of BaM6PI, the refined and validated BaM6PI model was used for docking. Hydrogen atoms were added to mimic the experimental pH conditions. To account for the solvation effect, the enzyme-substrate complex with a radius of 15 Å was solvated with water molecules. The structures of the protein and Zn^2+^ were energy minimized using the CHARMm force field present in DS 3.5. Catalytic Zn^2+^ was docked in the zinc-binding site to generate the holo enzyme. Next, the energy-minimized complex of the holo enzyme was used as the receptor for docking with its substrate, cyclic β-form of M6P [20]. For the comparative study, M6P was also docked in the K96Q mutant structure of BsM6PI (1QWR). To identify the differences in the molecular interactions for each ligand, 100 replicas were generated, and the docking poses were ranked based on the CDOCKER energy. The final pose of the docked substrate M6P was selected for both the BaM6PI model and the BsM6PI K96Q mutant model (Fig 6). M6P interacted with the enzyme through hydrogen bonds (green dotted lines), and the interacting amino acid residues in the binding pocket were conserved, with the only difference of Arg17/Lys15 (BaM6PI/BsM6PI).

Prior biochemical and structural analysis proposed a multistep mechanism for the isomerization of M6P to F6P: (1) binding of the ligand followed by ring opening, (2) isomerization, and (3) formation of the product, (4) closure of the ring followed by release of the product. The catalytically important residues involved are Gln98, Lys116, and Glu118. Binding of the ligand followed by ring opening is assisted by a zinc-bound water molecule and Gln98/Gln96 (BaM6PI/BsM6PI), and there is displacement of electrons between O1 and O5 oxygens, which induces breakage of the C1-O5 bond of M6P and generates the open form. In the M6P-docked BaM6PI model, the distances between C1-H_2_O, Gln98 (NE2)-O5, and Zn-H_2_O were 1.94, 2.22, and 1.61 Å, respectively. In the M6P-docked BsM6PI model, the distances between C1-H_2_O, Gln96 (NE2)-O5, and Zn-H_2_O were 3.29, 3.27, and 2.04 Å, respectively. During isomerization, there is an abstraction of a hydrogen atom on C2 of M6P by Lys116/Lys114 (BaM6PI/BsM6PI) in coordination with Glu118/Glu116 (BaM6PI/BsM6PI). In the product-formation step, protonation of C1 carbon by Lys116/Lys114 (BaM6PI/BsM6PI) forms F6P. In the M6P-docked BaM6PI model, the distance between Lys116 (NZ) and Glu118 (OE1) was 2.36 Å. In the M6P-docked BsM6PI model, however, the distance between Lys114 (NZ) and Glu116 (OE1) was 4.01 Å (Fig 6). Although the catalytic residues are highly conserved, the reason for the high catalytic efficiency of BaM6PI could be a more favorable distance between the catalytically important residues in comparison with BsM6PI. In the BaM6PI model, the hydrogen bonding is also in good coordination with the catalytically important residues for the transfer of the proton in coordination with the water and metal. The Arg17 residue provides additional hydrogen bonding with O4, stabilizing the substrate in the binding pocket.

## Conclusion

The *BaM6PI* gene encoding a M6PI was cloned from *B*. *amyloliquefaciens* and overexpressed in *E*. *coli*. The purified BaM6PI demonstrated the highest reported catalytic efficiency towards M6P to date, and displayed a 97% substrate conversion from M6P to F6P. In addition, BaM6PI functions at a broad pH range, with a relative activity > 85% between pH 5.0 and 9.0, demonstrating the potential for BaM6PI use in industrial applications. Further optimization of the bioconversion along with the mutant selection using protein engineering will be performed to improve the production of phosphorylated carbohydrate analogues with medicinal value.

## Supporting Information

S1 Fig Enzymatic cascade reactions for the preparation of sugar monophosphates. (TIF)

S2 Fig Proposed mechanism for the reversible isomerization of M6P to F6P. (TIF)

S3 Fig Structural alignment of the active site residues for the validity of BaM6PI docking. (TIF)

S4 Fig Determination of the molecular mass of BaM6PI by SDS-PAGE. (TIF)

S5 Fig Determination of the native molecular mass of *B*. *amyloliquefaciens* M6PI by gel filtration chromatography. (TIF)

S6 Fig Effect of various metal ions on BaM6PI activity. (TIF)

S7 Fig Effect of pH on the activity of BaM6PI (solid circle) and BsM6PI (open circle). (TIF)

S8 Fig Ramachandran plot of BaM6PI homology model. (TIF)

S1 Table List of strains to screen putative mannose-6-phosphate genes. (DOC)
