# Supplementary figures and images for: Characterization of a Mannose-6-Phosphate Isomerase from Bacillus amyloliquefaciens and Its Application in Fructose-6-Phosphate Production
Source: PLoS One. 2015 Jul 14;10(7):e0131585. doi: 10.1371/journal.pone.0131585 (PMC4718643; doi:10.1371/journal.pone.0131585)

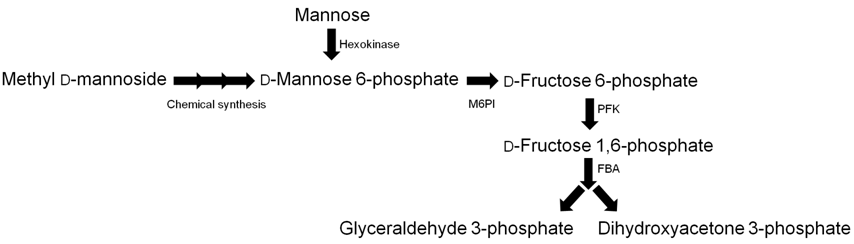

Supplement: S1 Fig — (TIF) [file pone.0131585.s001.tif]

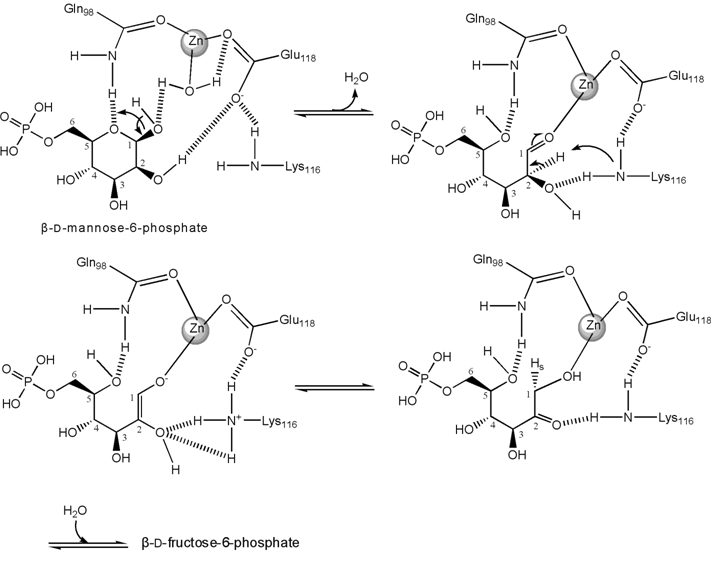

Supplement: S2 Fig — (TIF) [file pone.0131585.s002.tif]

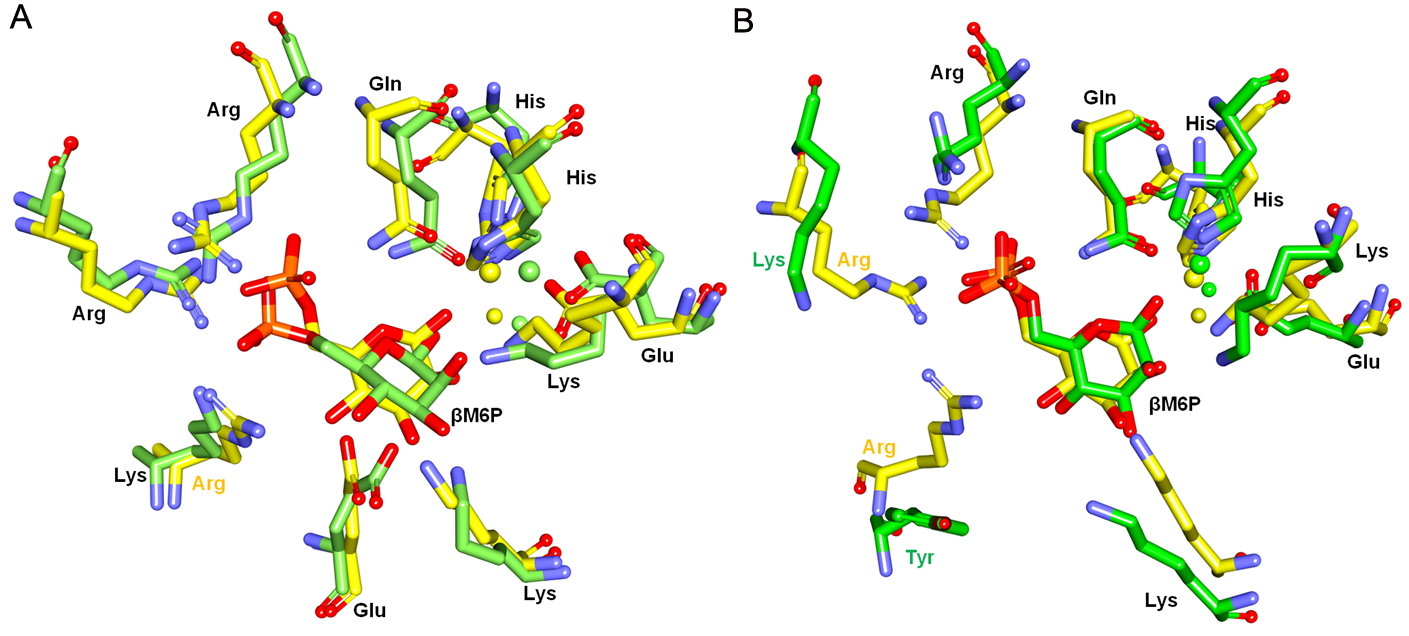

Supplement: S3 Fig — (TIF) [file pone.0131585.s003.tif]

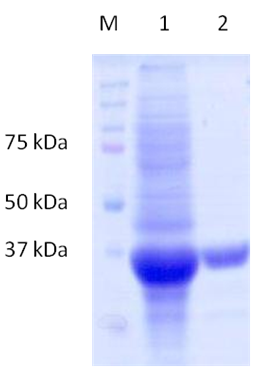

Supplement: S4 Fig — (TIF) [file pone.0131585.s004.tif]

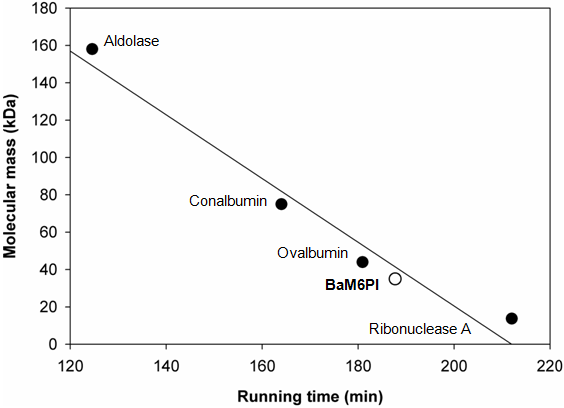

Supplement: S5 Fig — (TIF) [file pone.0131585.s005.tif]

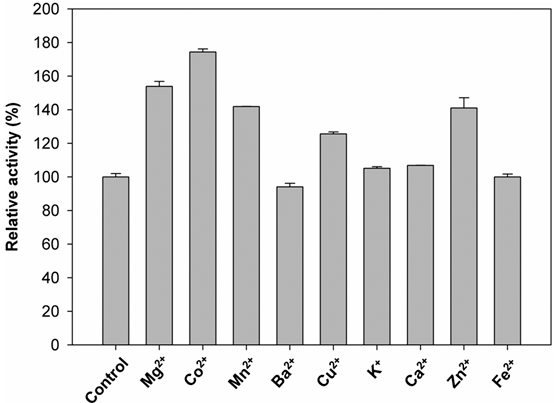

Supplement: S6 Fig — (TIF) [file pone.0131585.s006.tif]

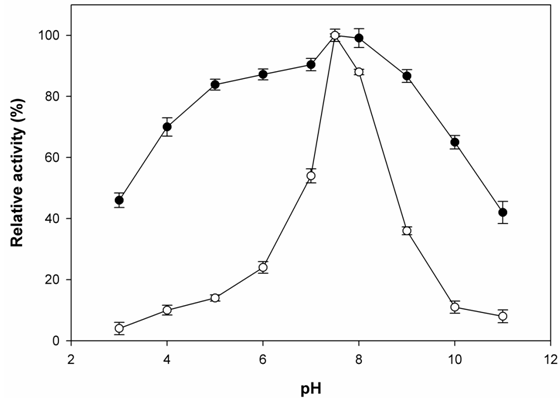

Supplement: S7 Fig — (TIF) [file pone.0131585.s007.tif]

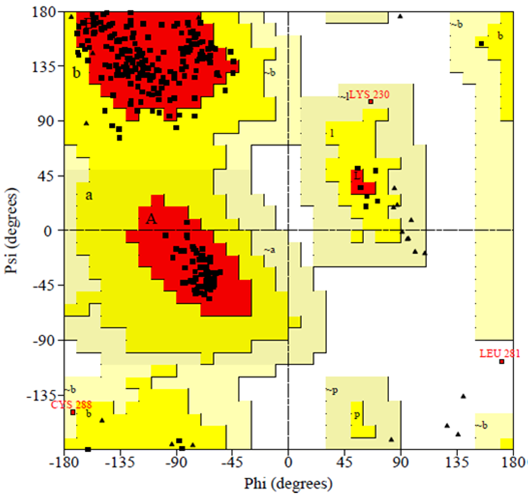

Supplement: S8 Fig — (TIF) [file pone.0131585.s008.tif]
