# Supplementary material for: Characterization of a Mannose-6-Phosphate Isomerase from Bacillus amyloliquefaciens and Its Application in Fructose-6-Phosphate Production
Source: PLoS One. 2015 Jul 14;10(7):e0131585. doi: 10.1371/journal.pone.0131585 (PMC4718643; doi:10.1371/journal.pone.0131585)
Supplement: S1 Table — (DOC) [file pone.0131585.s009.doc]

**S1 Table.** **List of strains to screen putative mannose-6-phosphate genes** having activity towards d-mannose-6-phosphate

| **S.N** | **Organisms** |
| --- | --- |
| 1 | *Corynebacterium efficiens* (DSM 44549) |
| 2 | *Geobacillus stearothermophilus* NUB3621 |
| 3 | *Listeria fleischmannii FSL S10-1203* |
| 4 | *Bacillus methylotrophicus* (KACC 13105) |
| 5 | *Streptomyces venezuelae* (ATCC 10712) |
| 6 | *Kitasatospora setae* (ATCC 33774) |
| 7 | *Saccharothrix espanaensis* (ATCC 51144) |
| 8 | *Lactobacillus plantarum* strain JDM1(KCTC 3108) |
| 9 | *Flavobacterium johnsoniae* (ATCC 17061) |
| 10 | *B. subtilis* strain 168 (ATCC 23857) |
| 11 | *Lactobacillus casei* (ATCC 334) |
| 12 | *Lactobacillus murinus* (KACC 12432) |
| 13 | *Streptococcus devriesei* (KACC 13876) |
| 14 | *Bacillus halodurans* (DSM 18197) |
| 15 | *Gluconobacter* *oxydans* strain 621H |
| 16 | *Geobacillus caldoxylosilyticus* (NBRC 107762) |
| 17 | *Bacillus licheniformis* (ATCC 14580) |
| 18 | *Streptococcus canis* (KACC 13819) |
| 19 | *Streptomyces avermitilis* (ATCC 31267) |
| 20 | *Streptococcus thermophilus* JIM 8232 |
| 21 | *Aneurinibacillus aneurinolyticus* (KCTC 3883) |
| 22 | *Amphibacillus xylanus* (DSM 6626) |
| 23 | *Oceanobacillus iheyensis* (KCTC 3954) |
| 24 | *Methylobacillus flagellatus* (ATCC 51484) |
| 25 | *Bacillus amyloliquefaciens* DSM7 (KCCM 40765) |
| 26 | *Lactobacillus salivarius* (DSM 20555) |
